# Complete genome sequence of the sulfate-reducing firmicute *Desulfotomaculum ruminis* type strain (DL^T^)

**DOI:** 10.4056/sigs.3226659

**Published:** 2012-12-11

**Authors:** Stefan Spring, Michael Visser, Megan Lu, Alex Copeland, Alla Lapidus, Susan Lucas, Jan-Fang Cheng, Cliff Han, Roxanne Tapia, Lynne A. Goodwin, Sam Pitluck, Natalia Ivanova, Miriam Land, Loren Hauser, Frank Larimer, Manfred Rohde, Markus Göker, John C. Detter, Nikos C. Kyrpides, Tanja Woyke, Peter J. Schaap, Caroline M. Plugge, Gerard Muyzer, Jan Kuever, Inês A. C. Pereira, Sofiya N. Parshina, Rizlan Bernier-Latmani, Alfons J.M. Stams, Hans-Peter Klenk

**Affiliations:** 1 Leibniz Institute DSMZ - German Collection of Microorganisms and Cell Cultures, Braunschweig, Germany; 2 Laboratory of Microbiology, Wageningen University, Wageningen, The Netherlands; 3 DOE Joint Genome Institute, Walnut Creek, California, USA; 4 Los Alamos National Laboratory, Bioscience Division, Los Alamos, New Mexico, USA; 5 Oak Ridge National Laboratory, Oak Ridge, Tennessee, USA; 6 HZI – Helmholtz Centre for Infection Research, Braunschweig, Germany; 7 Laboratory of Systems and Synthetic Biology, Wageningen University, Wageningen, The Netherlands; 8 Department of Aquatic Microbiology, Institute for Biodiversity and Ecosystem Dynamics, University of Amsterdam, Amsterdam, The Netherlands; 9 Department of Microbiology, Bremen Institute for Materials Testing, Bremen, Germany; 10 Instituto de Tecnologia Quimica e Biologica, Universidade Nova de Lisboa, Oeiras, Portugal; 11 Wingradsky Institute of Microbiology Russian Academy of Sciences, Moscow, Russia; 12 Ecole Polytechnique Federale de Lausanne, Lausanne, Switzerland

**Keywords:** anaerobic, motile, sporulating, mesophilic, sulfate-reducer, hydrogen sulfide, incomplete oxidizer, mixotrophic, CSP 2009, *Peptococcaceae*, *Clostridiales*

## Abstract

*Desulfotomaculum ruminis* Campbell and Postgate 1965 is a member of the large genus *Desulfotomaculum* which contains 30 species and is contained in the family *Peptococcaceae*. This species is of interest because it represents one of the few sulfate-reducing bacteria that have been isolated from the rumen. Here we describe the features of *D. ruminis* together with the complete genome sequence and annotation. The 3,969,014 bp long chromosome with a total of 3,901 protein-coding and 85 RNA genes is the second completed genome sequence of a type strain of the genus *Desulfotomaculum* to be published, and was sequenced as part of the DOE Joint Genome Institute Community Sequencing Program 2009.

## Introduction

Strain DL^T^ (= DSM 2154 = ATCC 23193 = NCIMB 8452) is the type strain of the species *Desulfotomaculum ruminis* [1], one out of currently 30 species with validly published names in the paraphyletic genus *Desulfotomaculum* [2,3]. Strain DL^T^ was initially isolated by G. S. Coleman in the 1950s from the rumen of hay-fed sheep [4]. Dissimilatory reduction of sulfate to sulfide in the rumen was first demonstrated by Lewis [5], who dosed fistulated sheep with sulfate and determined the amount of sulfide produced. As high amounts of sulfide may be toxic to animals, bacterial sulfate-reduction in ruminants was a concern due to the presence of sulfate in grass and hay. *D. ruminis* represented the first pure culture of a sulfate-reducing bacterium isolated from the rumen. The genus name was derived from the Latin words 'de', from, ‘sulfur’, sulfur, and 'tomaculum', a kind of sausage, meaning 'a sausage-shaped sulfate reducer' [2,6]. The species epithet is derived from the Latin word 'rumen', throat, first stomach (rumen) of a ruminant, meaning of a rumen [1,2]. Here, we present a summary classification and a set of features for *D. ruminis* strain DL^T^, together with the description of the complete genomic sequencing and annotation. The complete genome sequence of strain DL^T^ will provide valuable information for defining a more adequate description of the currently paraphyletic genus *Desulfotomaculum*.

### Classification and features

A representative genomic 16S rRNA sequence of *D. ruminis* DSM 2154^T^ was compared using NCBI BLAST [7,8] under default settings (e.g., considering only the high-scoring segment pairs (HSPs) from the best 250 hits) with the most recent release of the Greengenes database [9] and the relative frequencies of taxa and keywords (reduced to their stem [10]) were determined, weighted by BLAST scores. The most frequently occurring genera were *Desulfotomaculum* (88.3%), *Pelotomaculum* (7.9%), *Cryptanaerobacter* (2.8%) and *'Catabacter'* (1.0%) (60 hits in total). Regarding the four hits to sequences from members of the species, the average identity within HSPs was 99.1%, whereas the average coverage by HSPs was 86.1%. Regarding the 41 hits to sequences from other members of the genus, the average identity within HSPs was 93.2%, whereas the average coverage by HSPs was 90.7%. Among all other species, the one yielding the highest score was *Desulfotomaculum putei* (HM228397), which corresponded to an identity of 94.1% and an HSP coverage of 98.5%. (Note that the Greengenes database uses the INSDC (= EMBL/NCBI/DDBJ) annotation, which is not an authoritative source for nomenclature or classification.) The highest-scoring environmental sequence was EU307084 ('Changes microbial metabolic and along hydrogeochemically variable profile unsaturated horizon soil aggregate clone A Ac-2 12'), which showed an identity of 97.5% and an HSP coverage of 98.4. *D. ruminis* has not only been found in the rumen of animals, but also in other environments [11,12]. Therefore, the distribution of *D. ruminis* is not restricted to the rumen of animals. Hence, it is likely that this species enters the digestive tract of ruminants *via* food contaminated by spores.

**Figure 1:**
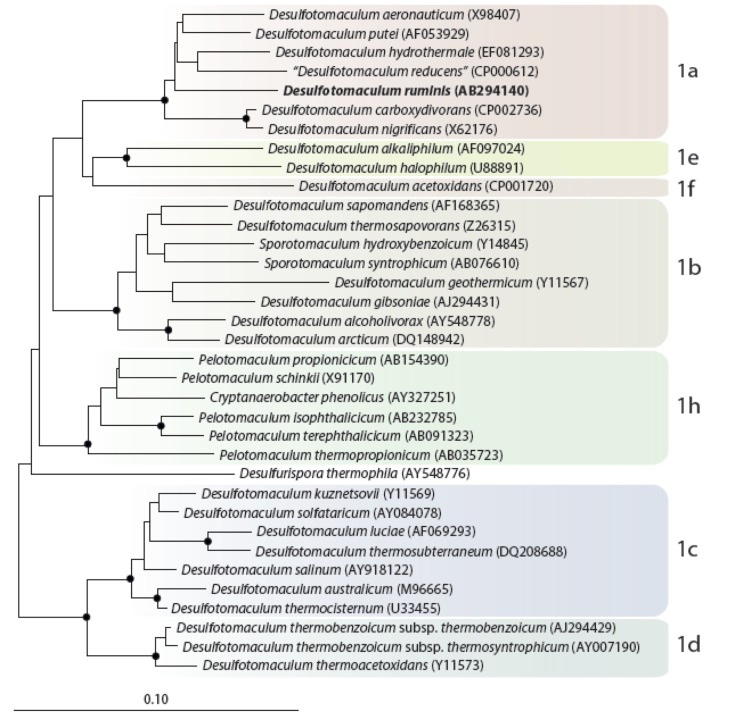
Neighbor-joining tree based on 16S rRNA sequences showing the phylogenetic affiliation of *Desulfotomaculum* and related species. *D. ruminis* is printed in bold type. The sequences of different *Thermotogales* were used as outgroup, but were pruned from the tree. Closed circles represent bootstrap values between 75 and 100%. The scale bar represents 2% sequences difference.

Figure 1 shows the phylogenetic neighborhood of *D. ruminis* in a 16S rRNA based tree of type strains. The sequences of the five 16S rRNA gene copies in the genome differ from each other by up to two nucleotides, and differ by up to three nucleotides from the previously published 16S rRNA sequence (Y11572), which contains three ambiguous base calls.

**Figure 2:**
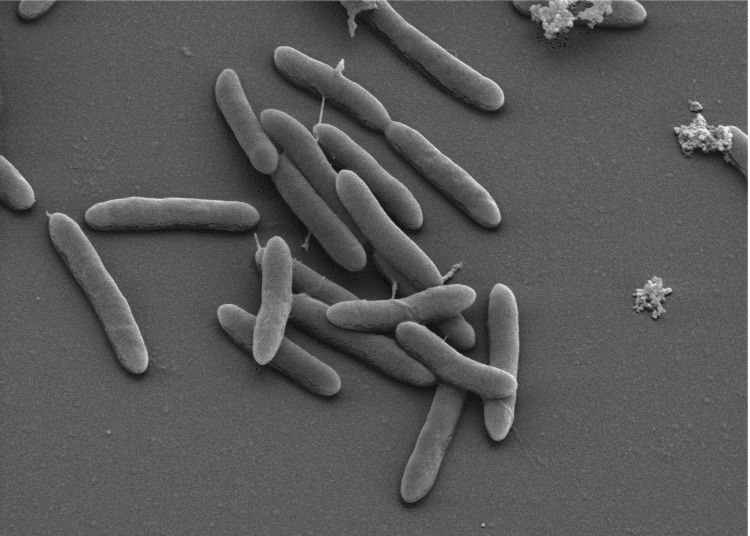
Scanning electron micrograph of *D. ruminis* DL^T^.

Cells of *D. ruminis* DL^T^ are slightly curved rods with rounded ends 2-6 µm in length and 0.5-0.7 µm in width (Table 1 and Figure 2) [1,4]. Cells stain Gram-negative and form oval subterminal spores that slightly swell the cells. A slight tumbling motility is due to peritrichous flagellation [1]. Strain DL^T^ grows optimally at 37°C, but not above 48°C [1]. The pH range for growth is 6.0-8.5 with an optimum between pH 6.0 and 7.0 [4]. *D. ruminis* strains are obligately anaerobic and can grow chemoheterotrophically with lactate, pyruvate, ethanol or alanine as well as mixotrophically with hydrogen or formate as electron donor and acetate as carbon source. In contrast to the distantly related *D. acetoxidans,* strains of *D. ruminis* oxidize substrates incompletely to acetate and cannot grow autotrophically [4]. Suitable electron acceptors are sulfate, thiosulfate and sulfite, but not elemental sulfur or nitrate [1,26]. Fermentative growth with pyruvate as sole substrate is also possible [26].

**Table 1:** Classification and general features of *D. ruminis* DL^T^ according to the MIGS recommendations [13] and the NamesforLife database [3].

MIGS ID	Property	Term	Evidence code
	Current classification	Domain *Bacteria*	TAS [14]
		Phylum *Firmicutes*	TAS [15-17]
		Class *Clostridia*	TAS [18,19]
		Order *Clostridiales*	TAS [20,21]
		Family *Peptococcaceae*	TAS [20,22]
		Genus *Desulfotomaculum*	TAS [1,6,20]
		Species *Desulfotomaculum ruminis*	TAS [1,20]
		Type strain DL	TAS [1]
	Gram stain	negative	TAS [1]
	Cell shape	rod-shaped	TAS [1]
	Motility	motile	TAS [1]
	Sporulation	sporulating	TAS [1]
	Temperature range	48°C is the upper limit	TAS [1]
	Optimum temperature	37°C	TAS [1]
	Salinity	not reported	
MIGS-22	Oxygen requirement	obligate anaerobe	TAS [1]
	Carbon source	acetate in combination with CO_2_ and a variety of other organic compounds	TAS [23]
	Energy metabolism	mixotrophic, heterotrophic	TAS [1,23]
MIGS-6	Habitat	rumen contents of sheep, fresh water, mud, sea water, soil	TAS [1]
MIGS-15	Biotic relationship	free-living	TAS [1]
MIGS-14	Pathogenicity	none	TAS [1]
	Biosafety level	1	TAS [24]
	Isolation	rumen of hay-fed sheep	TAS [1]
MIGS-4	Geographic location	Babraham, Cambridgeshire, UK	TAS [4]
MIGS-5	Sample collection time	1955 or before	TAS [4]
MIGS-4.1	Latitude	52.134	TAS [4]
MIGS-4.2	Longitude	0.206	TAS [4]
MIGS-4.3	Depth	not reported	
MIGS-4.4	Altitude	not reported	

Evidence codes - TAS: Traceable Author Statement (i.e., a direct report exists in the literature); NAS: Non-traceable Author Statement (i.e., not directly observed for the living, isolated sample, but based on a generally accepted property for the species, or anecdotal evidence). Evidence codes are from the Gene Ontology project [25].

### Chemotaxonomy

In cells of *D. ruminis* cytochromes of the *b*-type dominate [1], which is a typical trait of sulfate-reducing bacteria belonging to the genus *Desulfotomaculum*. Respiratory lipoquinones are also present and are comprised mainly of the menaquinone MK-7 and some small amounts of MK-6 [27]. The whole-cell fatty acid pattern of the type strain of *D. ruminis* was determined by Hagenauer *et al*. [26], who found a dominance of branched-chain iso- and anteiso-fatty acids in addition to unsaturated fatty acids, whereas saturated unbranched fatty acids were of less importance. The predominant fatty acids were: *iso*-C_17:1 c7_, *iso*-C_15:0_, *iso*-C_17:0_, C_17:0 cyc_ and C_16:0_. Although, in the study of Hagenauer *et al*. [26] a large amount of the extracted cellular fatty acids (37.3%) remained unidentified, the fatty acid pattern of *D. ruminis* can be clearly distinguished from other distantly related *Desulfotomaculum* species like *D. acetoxidans*, which has a pattern dominated by straight-chain saturated fatty acids, thus further illustrating the paraphyletic origin of this genus.

## Genome sequencing and annotation

### Genome project history

This organism was selected for sequencing on the basis of the DOE Joint Genome Institute Community Sequencing Program 2009 proposal 300132_795700 'Exploring the genetic and physiological diversity of *Desulfotomaculum* species'. The genome project is deposited in the Genomes OnLine Database (Gc01775) [28] and the complete genome sequence is deposited in GenBank (CP002780). Sequencing, finishing and annotation were performed by the DOE Joint Genome Institute (JGI). A summary of the project information is shown in Table 2.

**Table 2:** Genome sequencing project information

**MIGS ID**	**Property**	**Term**
MIGS-31	Finishing quality	Finished
MIGS-28	Libraries used	Three genomic libraries: two 454 pyrosequence standard library, one 454 PE library (9 kb insert size), one Illumina library
MIGS-29	Sequencing platforms	Illumina GAii, 454 GS FLX Titanium
MIGS-31.2	Sequencing coverage	193.0 × Illumina; 28.0 × pyrosequence
MIGS-30	Assemblers	Newbler version 2.3, Velvet 0.7.63, phrap version SPS - 4.24
MIGS-32	Gene calling method	Prodigal 1.4, GenePRIMP
	INSDC ID	CP002780
	Genbank Date of Release	October 12, 2011
	GOLD ID	Gc01775
	NCBI project ID	47605
	Database: IMG	650716033
MIGS-13	Source material identifier	DSM 2154
	Project relevance	Biotechnology, carbon cycle; sulfur cycle, metal precipitation

**Growth conditions and DNA isolation**

*D. ruminis* strain DL^T^, DSM 2154, was grown anaerobically in DSMZ medium 63 (*Desulfovibrio* medium) [29] at 37°C. DNA was isolated from 0.5-1.0 g of cell paste using Jetflex Genomic DNA Purification Kit (GENOMED 600100) following the manufacturer’s instructions, with a modified protocol for cell lysis (modification st/LALMP) as described in Wu *et al*. 2009 [30]. DNA is available through the DNA Bank Network [31].

### Genome sequencing and assembly

The genome was sequenced using a combination of Illumina and 454 sequencing platforms. All general aspects of library construction and sequencing can be found at the JGI website [32]. Pyrosequencing reads were assembled using the Newbler assembler (Roche). The initial Newbler assembly consisting of 74 contigs in one scaffold was converted into a phrap [33] assembly by making fake reads from the consensus, to collect the read pairs in the 454 paired end library. Illumina GAii sequencing data (1,651.9 Mb) was assembled with Velvet [34] and the consensus sequences were shredded into 1.5 kb overlapped fake reads and assembled together with the 454 data. The 454 draft assembly was based on 117.7 Mb 454 draft data and all of the 454 paired end data. Newbler parameters are -consed -a 50 -l 350 -g -m -ml 20. The Phred/Phrap/Consed software package [33] was used for sequence assembly and quality assessment in the subsequent finishing process. After the shotgun stage, reads were assembled with parallel Phrap (High Performance Software, LLC). Possible mis-assemblies were corrected with gapResolution [32], Dupfinisher [35], or sequencing cloned bridging PCR fragments with subcloning. Gaps between contigs were closed by editing in Consed, by PCR and by Bubble PCR primer walks (J.-F. Chang, unpublished). A total of 255 additional reactions were necessary to close gaps and to raise the quality of the finished sequence. Illumina reads were also used to correct potential base errors and increase consensus quality using a software Polisher developed at JGI [36]. The error rate of the completed genome sequence is less than 1 in 100,000. Together, the combination of the Illumina and 454 sequencing platforms provided 221-fold coverage of the genome. The final assembly contained 229,368 pyrosequence and 20,934,522 Illumina reads.

### Genome annotation

Genes were identified using Prodigal [37] as part of the Oak Ridge National Laboratory genome annotation pipeline, followed by a round of manual curation using the JGI GenePRIMP pipeline [38]. The predicted CDSs were translated and used to search the National Center for Biotechnology Information (NCBI) nonredundant database, UniProt, TIGR-Fam, Pfam, PRIAM, KEGG, COG, and InterPro databases. Additional gene prediction analysis and functional annotation was performed within the Integrated Microbial Genomes - Expert Review (IMG-ER) platform [39].

### Genome properties

**Figure 3:**
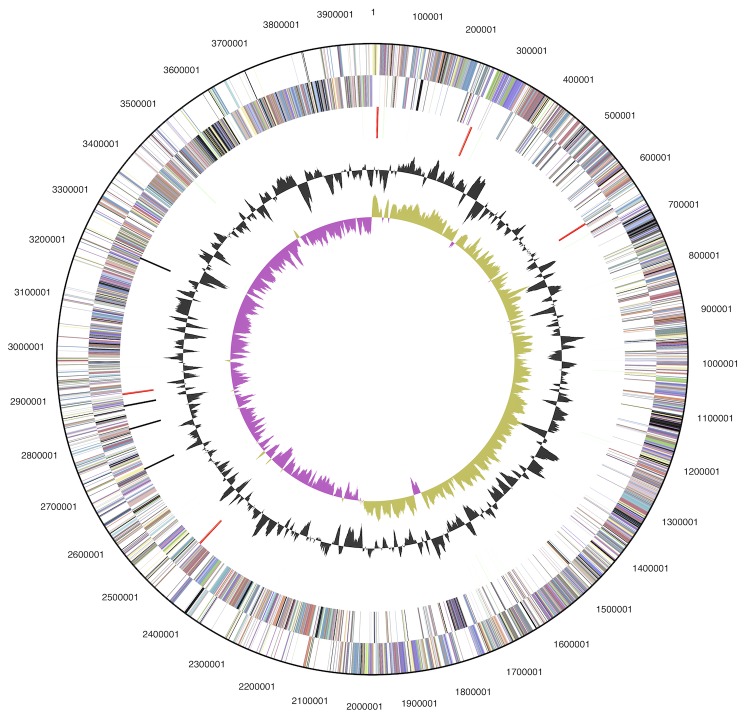
Graphical map of the chromosome. From outside to the center: Genes on forward strand (colored by COG categories), Genes on reverse strand (colored by COG categories), RNA genes (tRNAs green, rRNAs red, other RNAs black), GC content (black), GC skew (purple/olive).

The genome consists of one circular chromosome of 3,969,014 bp with a G+C content of 47.2% (Table 3 and Figure 3). Of the 3,986 genes predicted, 3,901 are protein-coding genes, and 85 are RNAs; 105 pseudogenes were also identified. The majority of the protein-coding genes (67.3%) were assigned with a putative function while the remaining ones were annotated as hypothetical proteins. The distribution of genes into COGs functional categories is presented in Table 4.

**Table 3:** Genome Statistics

**Attribute**	Value	% of Total^a^
Genome size (bp)	3,969,014	100.00%
DNA coding region (bp)	3,356,856	84.58%
DNA G+C content (bp)	1,875,083	47.24%
Number of replicons	1	
Extrachromosomal elements	0	
Total genes	3,986	100.00%
RNA genes	85	2.13%
rRNA operons	5	
Protein-coding genes	3,901	97.87%
Pseudo genes	105	2.63%
Genes with function prediction	2,682	67.29%
Genes in paralog clusters	2,015	50.55%
Genes assigned to COGs	2,897	72.68%
Genes assigned Pfam domains	3,047	76.44%
Genes with signal peptides	1,077	27.02%
Genes with transmembrane helices	1,016	25.49%
CRISPR repeats	11	

**Table 4:** Number of genes associated with the general COG functional categories

**Code**	**Value**	**%age**	**Description**
J	160	5.0	Translation, ribosomal structure and biogenesis
A	0	0.0	RNA processing and modification
K	293	9.1	Transcription
L	177	5.5	Replication, recombination and repair
B	1	0.0	Chromatin structure and dynamics
D	49	1.5	Cell cycle control, cell division, chromosome partitioning
Y	0	0.0	Nuclear structure
V	59	1.8	Defense mechanisms
T	246	7.7	Signal transduction mechanisms
M	161	5.0	Cell wall/membrane/envelope biogenesis
N	88	2.7	Cell motility
Z	0	0.0	Cytoskeleton
W	0	0.0	Extracellular structures
U	81	2.5	Intracellular trafficking, secretion, and vesicular transport
O	107	3.3	Posttranslational modification, protein turnover, chaperones
C	252	7.9	Energy production and conversion
G	155	4.8	Carbohydrate transport and metabolism
E	279	8.7	Amino acid transport and metabolism
F	65	2.0	Nucleotide transport and metabolism
H	147	4.6	Coenzyme transport and metabolism
I	54	1.7	Lipid transport and metabolism
P	154	4.8	Inorganic ion transport and metabolism
Q	36	1.1	Secondary metabolites biosynthesis, transport and catabolism
R	351	10.9	General function prediction only
S	297	9.3	Function unknown
-	1,089	27.3	Not in COGs

## Insights into the genome sequence

### Electron donor utilization

#### Chemoheterotrophic growth

*D. ruminis* is an incomplete sulfate reducer and can metabolize various substrates as carbon and energy sources, including organic acids, alcohols and amino acids, to acetate [1,6,40]. In the *D. ruminis* genome, numerous genes are present that encode aminotransferases belonging to class I and II (Desru_0552, Desru_1291, Desru_1826, Desru_1950, Desru_2322, Desru_2323, Desru_3729), class III (Desru_0350, Desru_0589, Desru_3742), class IV (Desru_1652), and class V (Desru_0021), which indicates that besides alanine, other amino acids might be substrates for this species. The oxidative deamination step of the amino acid degradation is probably catalyzed by an alanine dehydrogenase, which exists in two copies (Desru_0588 and Desru_2884) or a glutamate dehydrogenase (Desru_0556), confirming previous physiological studies [40].

Interestingly, a taurine degradation pathway was also detected in the annotated genome. In habitats that are depleted of sulfate, like rumen or freshwater sediments, the amino sulfonic acid taurine could represent a key substrate for *D. ruminis.* Taurine is widely distributed in animal tissue, especially the large intestine, and can be converted by a taurine-pyruvate aminotransferase (Desru_0589) to sulfoacetaldehyde, which in turn is cleaved by the enzyme sulfoacetaldehyde acetyltransferase (Desru_0590) into sulfite and acetyl-phosphate. Sulfite can then be used as electron acceptor and reduced to sulfide.

Several genes encoding dehydrogenases were detected that catalyze the oxidation of organic acids (e.g., lactate), or alcohols (e.g., ethanol). The main metabolic intermediate resulting from the oxidation of organic carbon compounds in incomplete oxidizing sulfate-reducing bacteria is pyruvate, which in *D. ruminis* can be degraded by the action of several enzymes: pyruvate dehydrogenase (Desru_0066 - 0067), pyruvate-ferredoxin oxidoreductase (Desru_0099 - 0102) and pyruvate-formate lyase (Desru_2143 and Desru_2090). The former two enzymes are decarboxylating and yield acetyl-CoA, CO_2_ and reducing equivalents, and the pyruvate-formate lyase produces acetyl-CoA and formate. The latter enzyme is preferentially used during fermentative metabolism, when pyruvate is the main carbon and energy source.

Acetyl-CoA is either used for biosynthetic reactions or can be transferred into acetyl-phosphate by a phosphate acetyltransferase. However, in the annotated genome of *D. ruminis* only a phosphate butyryltransferase (Desru_2256) was found. It could be that such enzymes are not specific for butanoyl-CoA and also use acetyl-CoA. An alternative pathway for the recycling of CoA could be catalyzed by the acetyl-coenzyme A synthetase (Desru_0761). This enzyme may use AMP and pyrophosphate that are formed in the ATP-sulfurylase and APS reductase reaction, respectively, for the production of acetate, CoA and ATP, though it is not clear if this acetyl-CoA synthetase is reversible. It may also be involved in the activation of acetate during mixotrophic growth. Acetyl-phosphate, which is also produced in the degradation of taurine, is converted to ATP and acetate by the enzyme acetate kinase (Desru_1705).

Three genes involved in the acetyl-CoA pathway were not detected. These are the acetyl-CoA synthase gene (*acsB*), and the genes for the large and small subunit of the corrinoid iron sulfur protein. Due to the absence of these genes, *D. ruminis* is unable to perform complete oxidation of organic compounds via the acetyl-CoA pathway, which is consistent with the published species description [41].

#### Mixotrophic growth

Based on genes identified within the genome sequence, data hydrogen, formate and carbon monoxide could be potential substrates for mixotrophic growth in *D. ruminis*. As observed for other clostridial sulfate reducers [11] the genome of *D. ruminis* encodes several copies of [FeFe] hydrogenases, including three copies of a trimeric NAD(P)-dependent hydrogenase (Desru_2398 - 2396, Desru_2393 - 2391, Desru_0516 - 0514), and two copies of a membrane-associated hydrogenase (Desru_3431-3433 and Desru_0447 - 0445) that includes a TAT signal peptide that is not predicted to be cleaved off using SignalP [42], but to form a transmembrane helix that anchors the protein to the extracytoplasmic side of the membrane. A monomeric [FeFe] hydrogenase (Desru_2180) and a hydrogenase encoding a PAS-sensing domain (Desru_2509), similar to HsfB [43] are also present. In addition, the utilization of hydrogen may also be catalyzed by a Ni,Fe hydrogenase encoded by the genes Desru_2370 - 2372. This enzyme is bound to the membrane by a cytochrome *b*, but seems to be cytoplasmic as no signal peptides are predicted. Two gene loci encoding formate dehydrogenases are located adjacently in the genome. Genes Desru_3012 - 3008 code for a membrane-associated enzyme in which the catalytic subunit is coded by three genes (Desru_3012 - 3010), as observed in other organisms. The first gene (Desru_3012) includes a TAT signal peptide, so the localization of the enzyme relative to the membrane will depend on whether this peptide associates with the catalytic subunit (Desru_3010) or not. The gene Desru_3011 encodes for the FeS domain of the catalytic subunit. The second formate dehydrogenase (Desru_3002-3005) is a tetrameric NAD(P)-dependent enzyme. Potential genes encoding the catalytic subunit of anaerobic-type carbon monoxide dehydrogenases (*cooS*) were identified at Desru_0859 and Desru_3320. However, no other CODH complex genes were found near either of the two *cooS* genes, except for *cooC* at Desru_0860.

While growth with hydrogen and formate with acetate as carbon source was confirmed in laboratory experiments, no growth was obtained with 5% (v/v) of carbon monoxide in the headspace gas atmosphere [23]. This is in contrast with the *cooS* present in the genome and brings into question the function of this gene in *D. ruminis*. In a study about the fermentation burst in *Desulfovibrio vulgaris* Hildenborough it was found that CO is produced at low levels during growth on pyruvate or lactate [41]. It was hypothesized that the catalytic subunit of carbon monoxide dehydrogenase could be involved in an internal metabolism or cycling of carbon monoxide. In *D. vulgaris*, the *cooS* gene (YP_011311.1) is downstream of a transcriptional regulator (YP_011310.1) and upstream of the *cooC* gene (YP_011312.1). This localization is similar to what we find in *D. ruminis*, Desru_0859, Desru_0858 and Desru_0860, respectively. Thus, carbon-monoxide dehydrogenases could play a role in the internal metabolism or cycling of carbon monoxide during growth of *D. ruminis* on organic acids. However, in contrast to *D. vulgaris*, no CO-induced hydrogenase (*coo)* is present in *D. ruminis*.

### Energy metabolism

The genome of *D. ruminis* encodes the full set of genes necessary for dissimilatory sulfate reduction as well as several membrane complexes, which deliver electrons from membrane electron carriers like menaquinol to cytosolic sulfate-reducing enzymes. The following genes encoding cytoplasmic enzymes for dissimilatory sulfate reduction were detected in the *D. ruminis* genome: Sulfate adenylyltransferase (ATP-sulfurylase, Desru_3378), adenosine-5'-phosphosulfate (APS) reductase (Desru_3376 - 3377) and dissimilatory sulfite reductase (Desru_0386 - 0387 and Desru_3723 - 3724). In *D. ruminis*, like in most sulfate reducers, ATP-sulfurylase and APS reductase are present as one copy. However, in contrast to most other sulfate reducers, *D. ruminis* contains two copies of the *dsrABD* genes. As observed in other *Desulfotomaculum* species, the alpha subunit of the APS reductase appears to be membrane-anchored.

The generation of a proton gradient across the cytoplasmic membrane is thought to be the main mechanism for generation of energy in sulfate-reducing bacteria. The coupling of the reduction of sulfate to sulfide, which occurs exclusively in the cytoplasm with a membrane-bound electron transport chain, and a vectorial proton transport across the membrane is still far from being understood. Electrons and protons required for the generation of a chemiosmotic gradient in Gram-positive sulfate reducers could be generated by the oxidation of small intermediate metabolites, like hydrogen, CO^-^ or formate at the cytoplasmic membrane. Several membrane-bound enzyme complexes were recently identified that could play a role in this process.

A membrane-bound pyrophosphatase (Desru_3593) could use the energy generated from the cleavage of pyrophosphate, which is formed in the activation of sulfate by the ATP-sulfurylase, for proton translocation.

The QmoAB complex (Desru_3374 - 3375) is suggested to play a role in donating electrons to the APS reductase [44], and the genes of both enzymes are located next to each other in the *D. ruminis* genome. The gene for the membrane subunit QmoC is absent, as in other clostridial sulfate reducers. The QmoA subunit is predicted to contain a signal peptide, but this likely forms a transmembrane helix, as it is still present in the mature protein [45], which indicates a localization at the inner surface of the cytoplasmic membrane. In Gram-negative sulfate-reducing bacteria, a transmembrane DsrMKJOP complex is conserved, which probably transfers electrons from the periplasmic space to the dissimilatory sulfite reductase. In Gram-positive sulfate reducers, only a truncated DsrMK complex seems to be present [46], which is encoded adjacent to a small soluble protein designated DsrC that is proposed to have a function in shuttling electrons from DsrK to the cytoplasmic DsrAB sulfite reductase [47]. In *D. ruminis* this enzyme system is encoded by the genes Desru_3734 - 3736, and a second copy of the *dsrMK* genes is present (Desru_2596 - 2597)

The two *c*-type cytochromes present in “*D. reducens”* and annotated as a nitrite reductase are absent in *D. ruminis*, which is consistent with the other *Desulfotomaculum* species sequenced to date (except *D. nigrificans*) [46].

An energy-conserving NADH-quinone oxidoreductase (Complex I, Desru_1808 - 1818 and Desru_0514 - 516) is present, which will couple NADH oxidation to proton translocation. Furthermore, a multimeric membrane-bound complex was identified at Desru_3260 - 3265, that belongs to the family of Ehr complexes (for energy-conserving hydrogenase related complex) first identified in *Geobacter* spp., but present in many microorganisms [48,49]. The subunits of Ehr complexes are related to subunits of complex I and the Ech energy conserving hydrogenases, but in most cases the cysteines binding the NiFe cluster are absent, so these complexes are not real hydrogenases. In *D. ruminis* EhrL (Desru_3264) the four Cys required to coordinate the catalytic center are present, so this complex may be a true energy-conserving hydrogenase. The proton gradient resulting from the above-mentioned reactions is used by a F_0_F_1_-type ATP synthase complex encoded by the genes Desru_3687 - 3694.

There are number of heterodisulfide reductases in the genome: three loci were identified which contained *hdr*A (Desru_0205, Desru_0212 and Desru_3375) and *hdr*B (Desru_3379 and Desru_2699) and *hdr*C-like (Desru_3380 and Desru_2700) heterodisulfide reductases. In addition, a fused *hdr*A with *mvh*D (methyl-viologen reducing hydrogenase delta subunit) was identified (Desru_3374).

### Comparative genomics

**Figure 4:**
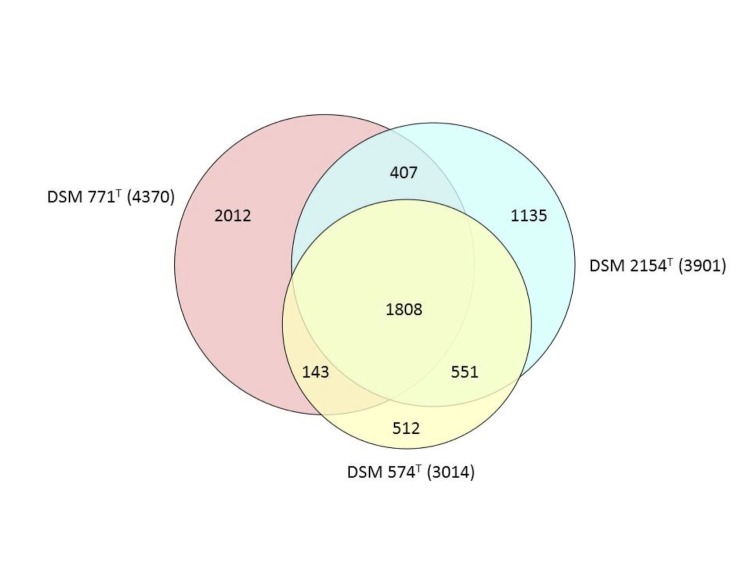
Venn diagram showing a comparison of three different *Desulfotomaculum* genomes, *D. ruminis* DSM 2154^T^, *D. acetoxidans* DSM 771^T^ and *D. nigrificans* DSM 564^T^. The number of overlapping protein genes is given inside the areas of the circles and the total number of derived protein sequences used for each strain is shown in parentheses. The figure was created using the program Venn diagram plotter available from the Pacific Northwest National Laboratory Software Distribution Center [51].

We analyzed the fraction of shared genes in three genomes of *Desulfotomaculum* species with validly published names. The genomes of *D. acetoxidans* [50] and *D. ruminis* [10] are complete, whereas the genome of the type species *D. nigrificans* is only available as a draft sequence. *D. nigrificans* has the smallest genome with 3,014 protein coding sequences. The resulting data are illustrated in the Venn diagram shown in [Figure 4]. The largest overlap is found between the strains *D. nigrificans* DSM 574^T^ and *D. ruminis* DSM 2154^T^, which share 2,359 homologous proteins corresponding to 78.3% of the DSM 574^T^ genes and 60.5% of the DSM 2154^T^ genes. Thus, a closer relationship between *D. ruminis* DSM 2154^T^ and *D. nigrificans* DSM 574^T^ than between *D. acetoxidans* DSM 771^T^ and *D. nigrificans* DSM 574^T^, as suggested by the 16S rRNA based phylogenetic tree, is confirmed by whole-genome data.

Figures 5A and 5B show the organization of *dsr*AB (A), *qmo*BA, *apr*AB and *hdr*BC (B) and neighboring genes for *D. ruminis*, “*D. reducens”* and *D. acetoxidans*. In Figure 5 *dsr*D is upstream of *dsr*AB in all three strains. However, no other neighboring genes are similar to each other. In contrast, Figure 5B shows remarkable homology in gene organization for the *apr*AB gene neighborhood for *D. ruminis* and “*D. reducens”*. Gene sequence is also very similar for that region (53-94% identity for the genes displayed including hypothetical proteins) which suggests horizontal gene transfer from a common ancestor. The *dsr*AB and *apr*BA proteins of *D. ruminis* are more closely related to “*D. reducens”* than to *D. acetoxidans* [Figure 6A and 6B]. This is in accordance with the 16SrRNA based phylogenetic tree and the whole-genome data.

**Figure 5A:**
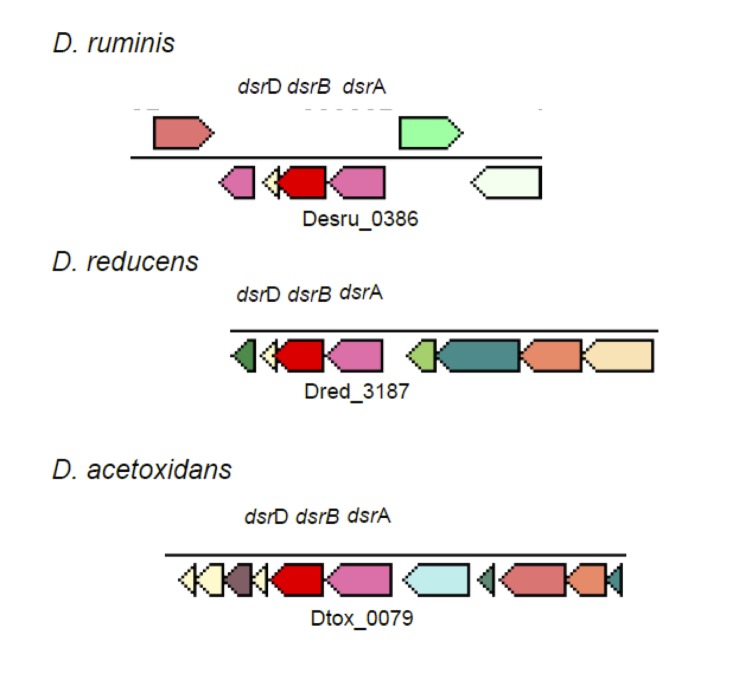
Organization of *dsr*AB and neighboring genes for three *Desulfotomaculum* species. Other genes are indicated by their locus tags.

**Figure 5B:**
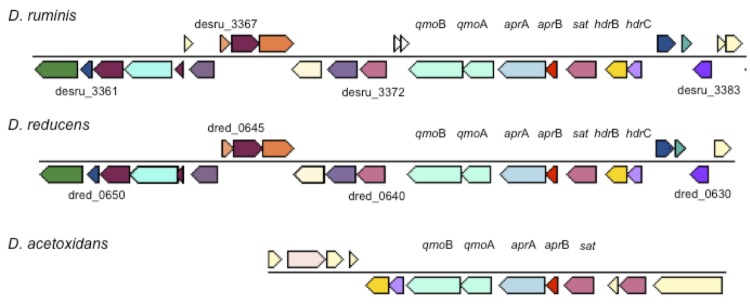
Organization of qmoBA, aprAB and hdrBC and neighboring genes for three *Desulfotomaculum* species. Other genes are indicated by their locus tags.

**Figure 6A:**
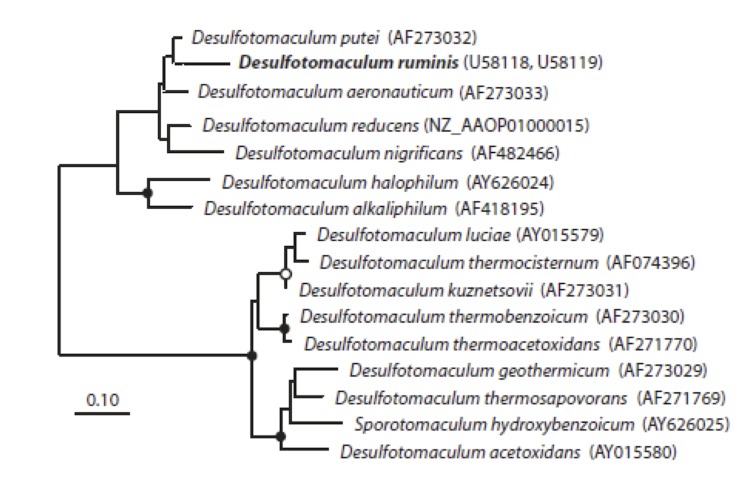
Phylogenetic tree of the dsrAB protein sequences. The trees (6A and 6B) were inferred from proteins sequences using RAxML (maximum-likelihood) in the software program ARB. The sequences of *Archaeoglobus fulgidus*, *A. profundus*, and *A. veneficus* were used as outgroup, but were pruned from the tree. The sequence of *D. ruminis* is written in bold. The black circles are bootstrap values between 100-75%, the white circles are values between 75-50%. The scale bar corresponds to 10% estimated sequence divergence.

**Figure 6B:**
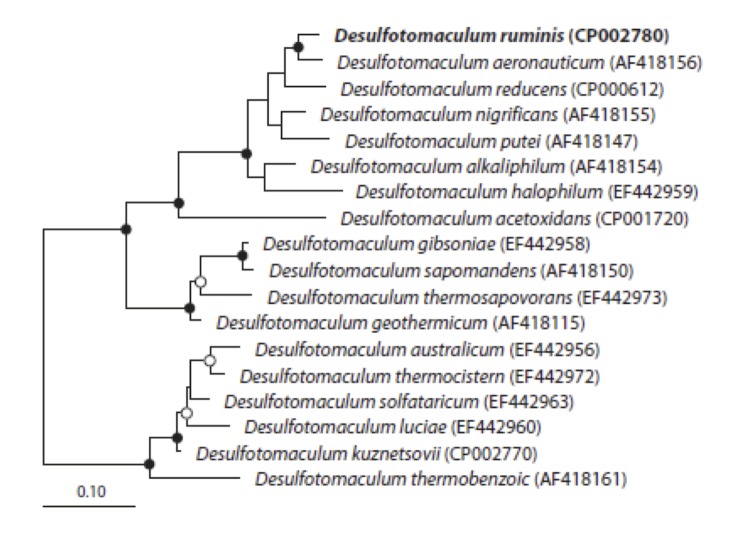
Phylogenetic tree of the aprBA protein sequence.
